# Retraction Note: MicroRNA-203 suppresses gastric cancer growth by targeting PIBF1/Akt signaling

**DOI:** 10.1186/s13046-017-0529-x

**Published:** 2017-05-03

**Authors:** Shao-Jun Chu, Ge Wang, Peng-Fei Zhang, Rui Zhang, Yan-Xia Huang, Yun-Min Lu, Wei Da, Qun Sun, Jing Zhang, Jin-Shui Zhu

**Affiliations:** 1 Department of Gerontology, Shanghai Jiao Tong University Affiliated Shanghai Sixth People’s Hospital, Shanghai, 200233 China; 2 Department of Gastroenterology, Shanghai Jiao Tong University Affiliated Shanghai Sixth People’s Hospital, Yishan Road No. 600, Shanghai, 200233 China

## Retraction

The authors are retracting this article [1] because it was brought to the Editor’s attention that two images appear to be similar to others within the same article and one image appears to be similar to an image in Zhang et al. [2], as detailed below:

- The two tubulin/HGC-27 bands in Fig. 4b [1] and the first two EGFR bands in the HGC-27 panel of Fig. 5b [1].
- The two tubulin/BGC-823 bands in Fig. 4b [1] and the second two EGFR bands in the HGC-27 panel of Fig. 5b [1].
- The four tubulin bands in the HGC-27 panel of Fig. 5b [1] and the four tubulin bands in Fig. 5a of Zhang et al. [2].

Peng-Fei Zhang and Yun-Min Lu have not responded to our correspondence about this retraction. All the other authors support this retraction.
